# High-risk adverse events in two types of single inhaler triple-therapy: a pharmacovigilance study based on the FAERS database

**DOI:** 10.3389/fphar.2024.1460407

**Published:** 2025-01-09

**Authors:** Zhishen Ruan, Chunbin Wang, Shasha Yuan, Yiling Fan, Bo Xu, Xiaodong Cong, Dan Li, Qing Miao

**Affiliations:** ^1^ Respiratory Department, Xiyuan Hospital of Chinese Academy of Chinese Medical Sciences, Beijing, China; ^2^ Dongying People’s Hospital (Dongying Hospital of Shandong Provincial Hospital Group), Dongying, Shandong, China; ^3^ Cardiovascular Department, Shuguang Hospital of Shanghai University of Traditional Chinese Medicine, Shanghai, China

**Keywords:** COPD, asthma, single-inhaler triple therapy, FAERS, adverse events

## Abstract

**Objective:**

Single inhaler triple therapy is widely used in Chronic Obstructive Pulmonary Disease (COPD) and asthma. This research aimed to analyze adverse events (AEs) associated with Budesonide/Glycopyrronium/Formoterol Fumarate (BUD/GLY/FOR) and Fluticasone Furoate/Umeclidinium/Vilanterol (FF/UMEC/VI).

**Methods:**

This is a cross-sectional study. BUD/GLY/FOR (2020Q3-2024Q3) and FF/UMEC/VI (2018Q1-2024Q3) report files were downloaded from the U.S. Food and Drug Administration’s (FDA) Adverse Event Reporting System (FAERS) database. We use reporting odds ratio (ROR), proportional reporting ratio (PRR), and Bayesian confidence propagation neural network (BCPNN) for disproportionality analysis. The aim was to explore associations between drugs and preferred term (PT) and system organ classification (SOC) levels. We focused on exploring the top 10 PTs of each drug’s BCPNN (IC) effect value and the PT of pneumonia.

**Results:**

16,355 AEs in BUD/GLY/FOR and 39,110 AEs in FF/UMEC/VI were extracted. Device use issues, oropharyngeal and vocal problems, pneumonia, *candida* infections, and urinary retention were the standard PTs present in drug leaflets. The risk of device use issues was higher in BUD/GLY/FOR, whereas the risk of pneumonia and *candida* infection in FF/UMEC/ VI had higher risk. Outside of the drug leaflets, both drugs were associated with a higher risk of AEs in vascular disorders. BUD/GLY/FOR group had a higher risk of AEs in body height decreased and hypoacusis. Notably, this study found an association between the above PTs and drugs, and the causal relationship needs to be verified by further longitudinal studies.

**Conclusion:**

Our study provides a preliminary exploration of the safety of clinical use of BUD/GLY/FOR and FF/UMEC/VI, and clinicians should be alert to potential adverse effects.

## Introduction

Chronic Obstructive Pulmonary Disease (COPD) and asthma are two prevalent respiratory diseases that significantly affect patients’ respiratory function and quality of life. Inhaled medications include long-acting beta2-agonists (LABA), long-acting muscarinic antagonists (LAMA), and inhaled glucocorticoids (ICS). This combination of drugs can act in concert through different mechanisms to improve respiratory symptoms, reduce the risk of exacerbations, and improve patients’ quality of life. In recent years, the research progress of inhaler triple therapy (LABA + LAMA + ICS) as an essential treatment for COPD and asthma has attracted much attention.

GOLD 2024 indicates triple therapy (LABA + LAMA + ICS) for patients at high risk for acute exacerbation of COPD who are poorly controlled on duo therapy (LABA + LAMA/LABA + ICS) or have eosinophils ≥300/ul (GOLD Report, 2024). The risk of acute exacerbation, risk of hospitalization, and risk of death were significantly lower in the triple therapy group of COPD patients compared with the duo therapy group (Martinez et al., 2021; Lipson et al., 2018; Lipson et al., 2020). Although there was an increased risk of pneumonia, triple therapy had a better benefit-risk ratio (Lee et al., 2019; Dransfield et al., 2021). GINA 2024 states that inhaler triple therapy may be considered for adult patients whose disease is not controlled with low to moderate doses of ICS and LABA (Reports, 2024). Studies have found that asthma patients in the triple therapy group exerted better results in improving lung function and reducing the number of acute exacerbations compared to the duo therapy group (Lee et al., 2021; Virchow et al., 2019). Several studies involving China, Spain, and the United Kingdom have shown that triple therapy is more cost-effective than single- or two-drug therapy (Zhou et al., 2021; Paly et al., 2022; Kendall et al., 2023). Patient compliance was improved with the application of a single inhaler compared to multi-inhaler triple therapy (Mannino et al., 2022).

Budesonide/Glycopyrronium/Formoterol Fumarate (BUD/GLY/FOR) and Fluticasone Furoate/Umeclidinium/Vilanterol (FF/UMEC/VI) are the two most common single-inhaler triple therapy. Regarding timing, FF/UMEC/VI was approved to begin marketing in the United States in September 2017 (FDA, 2017), followed by the European Union and China. BUD/GLY/FOR has been available in the U.S. since July 2020 (FDA, 2020) and has since been launched in Japan, China, and the European Union. Two meta-analyses suggest that BUD/GLY/FOR is comparable to other triple inhalations regarding acute exacerbations of COPD and improvement in lung function (Ferguson et al., 2020; Rogliani et al., 2022). Another study found FF/UMEC/VI superior to other triple-therapies for annual acute exacerbations and symptoms of COPD (Ismaila et al., 2022). There are few randomized controlled studies of BUD/GLY/FOR in the treatment of asthma, and even fewer studies comparing the efficacy of BUD/GLY/FOR with FF/UMEC/VI.

Similarly, there is a paucity of studies on BUD/GLY/FOR versus FF/UMEC/VI adverse drug events (AEs), and our study focused on exploring and comparing the AEs of the two drugs. Although clinical trials are essential in identifying drug AEs, certain AEs are not easily detected, thus affecting drug efficacy and patient prognosis (Feagins et al., 2019). This study’s purpose was to determine the AEs related to FF/UMEC/VI and BUD/GLY/FOR through the U.S. Food and Drug Administration’s (FDA) Adverse Event Reporting System (FAERS) to provide real-world evidence of potential AEs for single-inhaler triple therapy.

## Methods

### Data sources

We performed this real-world pharmacovigilance study based on FAERS. The FAERS database is an FDA adverse event reporting system that includes information on AE and drug medication errors (Sakaeda et al., 2013). The source of data for the FAERS database is primarily reports from within the United States, but also includes some international reports from multinational companies. FAERS is updated quarterly, and AE report data comes from physicians, consumers, or forms submitted by drug manufacturers (Matera et al., 2024). When an AE occurs, the appropriate person (e.g., physician or patient) submits a report to FAERS. When submitting a report, the patient may choose to remain anonymous or provide limited information. The database’s high sample size and broad range of AE reports make it the best way to explore rare AEs (Silberstein et al., 2023).

We downloaded the American Standard Code for Information Interchange (ASCII) reporting files for drugs in the FAERS. Considering the time to market of the drug and the number of AEs, we download the BUD/GLY/FOR reporting files for Q3 2020 through Q3 2024 and the FF/UMEC/VI reporting files for Q1 2018 through Q3 2024. The data were processed using R4.2.3.

### Data extraction and analysis

Remove duplicate items from the report. The latest report was retained for information with the same caseid number in the DEMO table. The Medex_UIMA_1.8.3 system standardized drug names to extract reports with BUD/GLY/FOR as the primary drug and reports with FF/UMEC/VI as the primary drug. The specific names of the medications screened are shown in Supplementary Tables S1, S2.

The Medical Dictionary of Regulatory Activities (MedDRA-v24) is a detailed collection of standardized medical terminology used to harmonize medication AEs (Sharma and Kumar, 2022; Javed and Kumar, 2024). The hierarchical structure of MedDRA consists of Low-Level Term (LLT), Preferred Term (PT), High-Level Term (HLT), Higher-Level Group Term (HLGT), and Systems and Organs Classification (SOC) (Brown, 2003). We coded and categorized the signals using the most commonly used PTs and SOCs.

Disproportionality analysis is a method to address the presence of asymmetry in pharmacovigilance analysis (van Puijenbroek et al., 2002). We used three types of disproportionality analyses, including reported odds ratio (ROR), proportional reporting ratio (PRR), and Bayesian confidence propagation neural network (BCPNN). ROR and PRR show better specificity for detecting early adverse reaction signals, whereas BCPNN has a more vital ability to detect rare signals (Singh, 2015; Zou et al., 2024). All of the above algorithms are based on a 2 × 2 list of columns (Table 1), while the formulas and thresholds for the three methods are shown in Table 2.

**TABLE 1:** Four grid table.

	Drug-related AEs	Non-drug-related AEs	Total
Drug	a	b	a + b
Non-drug	c	d	c + d
Total	a + c	b + d	N = a + b + c + d

**TABLE 2:** ROR, PRR, and BCPNN methods, formulas, and thresholds.

Method	Formula	Threshold
ROR	$\text{ROR}=\frac{a\;/\;c}{b\;/\;d}$	a ≥ 3 (lower limit) > 1
	$SE\left(lnROR\right)=\sqrt{\frac{1}{a}+\frac{1}{b}+\frac{1}{c}+\frac{1}{d}}$	
	$95\%CI=e^{\ln\left(ROR\right)\pm 1.96se}$	
PRR	$\text{PRR}=\frac{a\;/\;\left(a+b\right)}{c\;/\;\left(c+d\right)}$	a ≥ 3 (lower limit) > 1 and χ^2^ > 4
	$SE\left(lnPRR\right)=\sqrt{\frac{1}{a}-\frac{1}{a+b}+\frac{1}{c}-\frac{1}{c+d}}$	
	$95\%CI=e^{\ln\left(PRR\right)\pm 1.96se}$	
BCPNN	$\text{IC}=\log_{2}\frac{p\left(x,y\right)}{p\left(x\right)p\left(y\right)}=\log_{2}\frac{a\left(a+b+c+d\right)}{\left(a+b\right)\left(a+c\right)}$	IC025 > 0
	$E\left(\text{IC}\right)=\log_{2}\frac{\left(a+\gamma 11\right)\left(a+b+c+d+\alpha \right)\left(a+b+c+d+\beta \right)}{\left(a+b+c+d+\gamma \right)\left(a+b+\alpha 1\right)\left(a+c+\beta 1\right)}$	
	$V\left(\text{IC}\right)=\frac{1}{{\left(\ln2\right)}^{2}}\left[\frac{\left(a+b+c+d\right)-a+\gamma -\gamma 11}{\left(a+\gamma 11\right)\left(1+a+b+c+d+\gamma \right)}+\frac{\left(a+b+c+d\right)-\left(a+b\right)+a-\alpha 1}{\left(a+b+\alpha 1\right)\left(1+a+b+c+d+\alpha \right)}+\frac{\left(a+b+c+d+\alpha \right)-\left(a+c\right)+\beta -\beta 1}{\left(a+b+\beta 1\right)\left(1+a+b+c+d+\beta \right)}\right]$	
	$\gamma =\gamma 11\frac{\left(a+b+c+d+\alpha \right)\left(a+b+c+d+\beta \right)}{\left(a+b+\alpha 1\right)\left(a+c+\beta 1\right)}$	
	$\text{IC}-2\text{SD}=E\left(\text{IC}\right)-2\;\sqrt{V\left(\text{IC}\right)}$	

First, after removing duplicates from the data, we extracted adverse events regarding BUD/GLY/FOR and FF/UMEC/VI. We then plotted line graphs based on the number of AEs for both drugs with each quarter. We calculated the number of AEs, PRR, ROR, and IC values for BUD/GLY/ for or FF/UMEC/VI under each system based on system organ classification (SOC).

Then, at the preferred term (PT) level, we listed adverse events for both drugs in order of the number of adverse events. We classified “device use issue,” “wrong technique in product usage process,” and “product use issue” as “device use issue” to see the incidence of device use problems for both drugs. We also conducted subgroup analyses at the PT and SOC levels based on age, gender, and reporting countries subgroups.

At the PT level, we also showed the signal strength of each AE, sorted by IC effect size. We excluded two types of PTs, medication-related illnesses and equipment use problems, focusing on the top 10 AEs in terms of signal intensity for each medication and one AE, pneumonia (the AE we were interested in). In addition, PTs present outside the drug leaflets were also analyzed and compared. In addition, we summarized time-scan plots of safety signals reflecting trends in drug-AE pairings based on IC value. Stable associations between drugs and AEs were demonstrated when the IC values for each year in the time-scan plots were greater than zero. PTs with at least 2 years of data were selected for analysis.

## Results

### Descriptive characteristics

**FIGURE 1:**
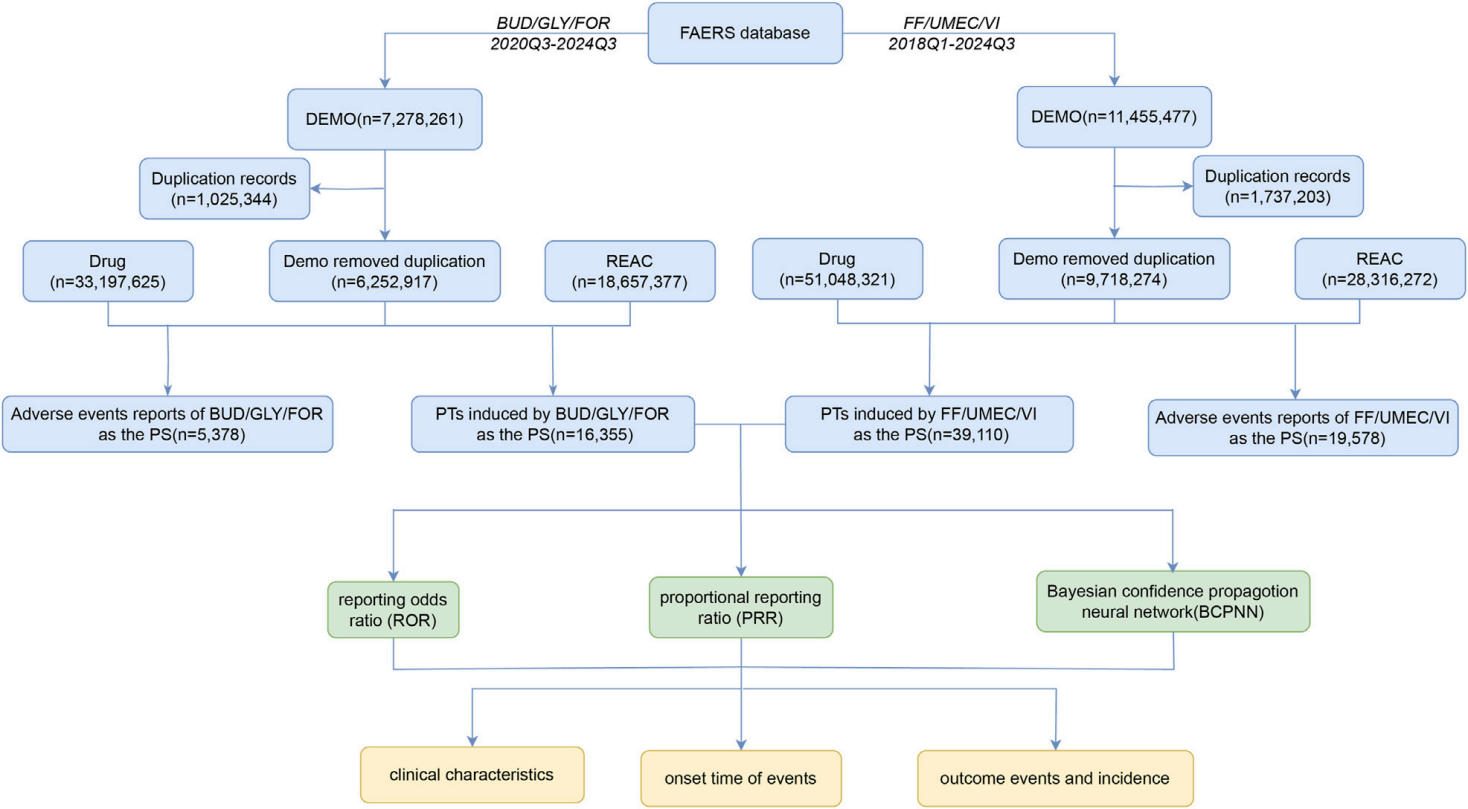
Flowchart of the study.

**FIGURE 2:**
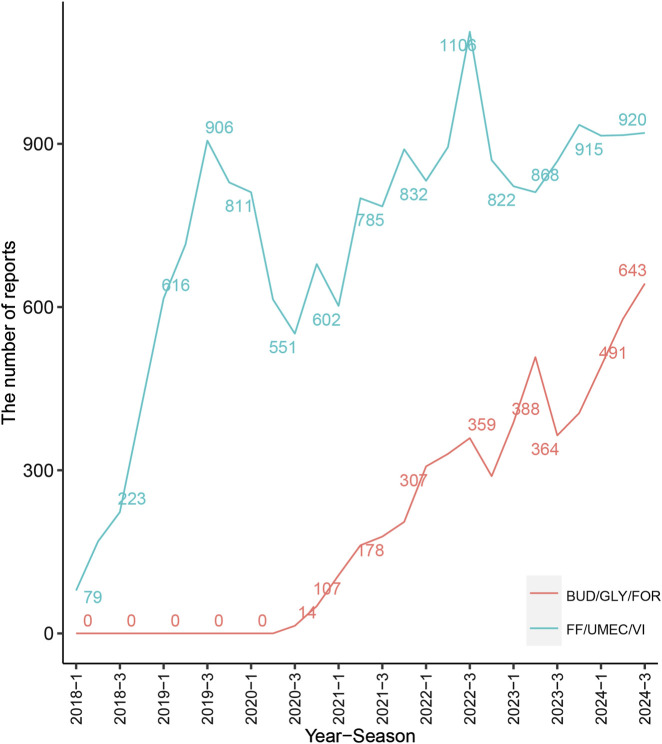
Number of reported cases of BUD/GLY/FOR and FF/UMEC/VI.

Figure 1 shows the screening process for the two drug AEs. First, 10276831 AE reports (2020Q3-2024Q3) and 6099615 AE reports (2018Q1-2024Q3) were collected from the FAERS database, respectively. After removing duplicate records, 5378 AE reports and 16,355 AEs were collected for BUD/GLY/FOR, and 19,578 AE reports and 39,110 AEs were collected for FF/UMEC/VI. Figure 2 shows the change in peaks per AE report, with FF/UMEC/VI having the highest percentage of AE reports in 2022 Q3 (1106,6.76%) and BUD/GLY/FOR having the highest number of AE reports in 2024 Q3 (643, 11.96%).

Table 3 shows the baseline information of the reporting population for both drug classes. Among them, the number of AE reports against FF/UMEC/VI and BUD/GLY/FOR was 9,079 (46.37%) and 2,443 (45.43%) in females, and AE reports were 6,361 (32.49%) and 1953 (36.31%) in males. In terms of age, apart from the fact that most of the data were missing, the 66–79 age group reported the highest proportion of AEs in the remaining data. Most AE reports originated from consumers (BUD/GLY/FOR, 74.17%; FF/UMEC/VI, 86.56%). In addition, the leading country for AE reporting is the United States, followed by Japan and China, and the main sources of FF/UMEC/VI reporting also involve Canada and the United Kingdom.

**TABLE 3:** Characterization of AE reports in BUD/GLY/FOR and FF/UMEC/VI.

	BUD/GLY/FOR	FF/UMEC/VI
Number of adverse events reported	5,378	19,578
Number of adverse events	16,355	39,110
Sex		
Female	2,443 (45.43)	9,079 (46.37)
Male	1953 (36.31)	6,361 (32.49)
Unknow	982 (18.26)	4,138 (21.14)
Age		
<18	4 (0.07)	14 (0.07)
18∼39	24 (0.45)	293 (1.50)
40∼65	506 (9.41)	1,152 (5.88)
66∼79	1,240 (23.06)	2,417 (12.35)
≥80	423 (7.87)	1,107 (5.65)
unknow	3,181 (59.15)	14,595 (74.55)
Reporter		
Consumer	3,989 (74.17)	16,946 (86.56)
Physician	712 (13.24)	1,268 (6.48)
Pharmacist	568 (10.56)	1,198 (6.12)
Unknow	109 (2.03)	27 (0.14)
Other health-professional	—	139 (0.71)
Reported countries (top five)		
United States	4,930 (91.67)	16,823 (85.93)
Other	221 (4.11)	243 (1.24)
Canada	—	1,659 (8.47)
Japan	165 (3.07)	281 (1.44)
China	62 (1.15)	52 (0.27)
United Kingdom	—	162 (0.83)
Year		
2018		891 (4.55)
2019		3,066 (15.66)
2020	64 (1.19)	2,655 (13.56)
2021	652 (12.12)	3,077 (15.72)
2022	1,285 (23.89)	3,702 (18.91)
2023	1,665 (30.96)	3,436 (17.55)
2024	1712 (31.83)	2,751 (14.05)

### Disproportionality analyses

Supplementary Table S3 shows the SOCs associated with BUD/GLY/FOR and FF/UMEC/VI. “Respiratory, thoracic and mediastinal disorders (BUD/GLY/FOR: 3,246, 19.85%; FF/UMEC/VI: 8,613, 22.02%),” “Injury, poisoning and procedural complications (BUD/GLY/FOR: 5,651, 34.55%; FF/UMEC/VI: 10,073, 25.76%),” “Eye disorders (BUD/GLY/FOR: 452, 2.76%; FF/UMEC/VI: 857, 2.19%)” were the three SOCs that conformed to the study algorithms. “Ear and labyrinth disorders” in BUD/GLY/FOR (91 cases, 0.56%) and “Infections and infestations” in FF/UMEC/VI (2,669 cases, 6.82%) fit the study algorithms. In addition, Supplementary Table S4 shows the number of AEs grouped by sex, age, and reporting countries at the SOC level.

Supplementary Table S5 shows that at the PT level, sorted by the number of AEs, we extracted a total of 882 PTs in both drugs. In addition to drug-related diseases, the number of signals of device misuse is very high. The results revealed that a higher percentage of patients had “device use issues” with BUD/GLY/FOR (2,430, 14.86%) compared to FF/UMEC/VI (2,314, 5.92%). Supplementary Table S5 shows that 320 PTs met the criteria of the study algorithm. After sorting by IC values, there were 141 PTs associated with BUD/GLY/FOR and 259 PTs associated with FF/UMEC/VI.

As shown in Table 4, a total of 17 PTs belonging to 7 socs were screened based on the top 10 highest IC values for each drug. In Respiratory, thoracic, and mediastinal disorders, the pharyngeal and vocal folds category of PTs were pharyngeal erythema (BUD/GLY/FOR: N = 9, 0.055%, ROR = 17.57, IC = 4.11; FF/UMEC/VI: N = 6, 0.015%, ROR = 4.79, IC = 2.25), vocal cord disorder (BUD/GLY/FOR: N = 9, 0.055%, ROR = 22.1, IC = 4.43; FF/UMEC/VI: N = 10, 0.026%, ROR = 9.81, IC = 2.42), dysphonia (BUD/GLY/FOR: 1.20%, N = 197, ROR = 12.98, IC = 3.48; FF/UMEC/VI: N = 751, 1.92%, ROR = 20.45, IC = 4.32), aphonia (BUD/GLY/FOR: N = 52, 0.32%, ROR = 13.64, IC = 3.75; FF/UMEC/VI: N = 116, 0.30%, ROR = 12.65, IC = 3.64). FF/UMEC/VI also had risks of vocal cord dysfunction (N = 10, 0.026%, ROR = 16.28, IC = 4.00), paranasal sinus inflammation (N = 4, 0.010%, ROR = 18.42, IC = 4.17), increased viscosity of bronchial secretion (N = 6, 0.015%, ROR = 15.89, IC = 3.96), which were not present in BUD/GLY/FOR. On Gastrointestinal disorders, the PTs common to both drugs were oral mucosal exfoliation (BUD/GLY/FOR: N = 3, 0.018%, ROR = 10.06, IC = 3.32; FF/UMEC/VI: N = 3, 0.0076%, ROR = 3.67, IC = 1.87). Coated in mouth (N = 14, 0.036%, ROR = 33.74, IC = 5.01) and tongue coated (N = 16, 0.041%, ROR = 19.38, IC = 4.24) were the top ten PTs with IC values unique to FF/UMEC/VI.

**TABLE 4:** BUD/GLY/FOR and FF/UMEC/VI AE signal strength reporting at preferred term (PT) levels.

SOCs/PTs	BUD/GLY/FOR					FF/UMEC/VI				
	N	PRR	ROR (95%CI^a^)	IC (IC025)	chisq	N	PRR	ROR (95%CI^a^)	IC (IC025)	chisq
Respiratory, thoracic and mediastinal disorders										
Pharyngeal erythema	9	17.57	17.57 (9.02)	4.11 (3.21)	138.47	6	4.79	4.79 (2.14)	2.25 (1.18)^a^	17.89
Vocal cord disorder	9	22.02	22.01 (11.3)	4.43 (3.53)	177.11	10	9.81	9.81 (5.24)	3.28 (2.42)^a^	78.06
Vocal cord dysfunction	—					10	16.29	16.28 (8.69)	4.00 (3.13)	140.3
Dysphonia	197	13.13	12.98 (11.32)	3.68 (3.48)	2,155.67	751	20.83	20.45 (18.91)	4.32 (4.21)	13,521.85
Aphonia	52	13.68	13.64 (10.37)	3.75 (3.36)	601.9	116	12.69	12.65 (10.6)	3.64 (3.37)^a^	1,223.89
Paranasal sinus inflammation	—					4	18.42	18.42 (6.78)	4.17 (2.88)	64.26
Increased viscosity of bronchial secretion	—					6	15.89	15.89 (7.11)	3.96 (2.88)	81.92
Gastrointestinal disorders										
Oral mucosal exfoliation	3	10.06	10.06 (3.23)	3.32 (1.90)	24.26	3	3.67	3.67 (1.18)	1.87 (0.45)^a^	5.8
Coating in mouth	—					14	33.75	33.74 (19.88)	5.01 (4.27)	424.96
Tongue coated	—					16	19.38	19.38 (11.87)	4.24 (3.54)	271.59
Infections and infestations										
*Candida* infection	77	14.81	14.75 (11.89)	3.87 (3.54)	974.49	308	24.51	24.33 (21.63)	4.56 (4.39)	6,668.1
Oral candidiasis	33	10.23	10.21 (7.32)	3.34 (2.85)	271.68	148	19.28	19.21 (16.42)	4.23 (3.99)	2,488.85
Pneumonia	134	1.65	1.64 (1.37)	0.71 (0.47)^b^	33.59	738	3.59	3.54 (3.27)	1.82 (1.71)^b^	1,343.63
Renal and urinary disorders										
Urine flow decreased	5	11.47	11.47 (4.75)	3.51 (2.34)	47.29	19	17.76	17.75 (11.31)	4.12 (3.48)	293.1
Investigations										
Body height decreased	38	18.52	18.48 (13.51)	4.19 (3.73)	618.26	—				
Injury, poisoning and procedural complications										
Lip injury	4	10.83	10.83 (4.06)	3.42 (2.15)	35.35					
Foreign body in mouth						5	54.78	54.77 (22.23)	5.67 (4.47)	245.37

^a^ represents PTs, that are not in the top 10 of effect values, but are significant in all three algorithms.

^b^ represents PTs, that are not in the top 10 of effect values, but are the focus of attention in this article./: Indicates non-compliance with any of the three algorithms. a: 95% lower bound of confidence interval.

In Infections and infestations, *candida* infection (BUD/GLY/FOR: N = 77, 0.47%, ROR = 14.75, IC = 3.87; FF/UMEC/VI: N = 308, 0.79%, ROR = 24.33, IC = 4.56) and oral candidiasis (BUD/GLY/FOR: N = 33, 0.20%, ROR = 10.21, IC = 3.34; FF/UMEC/VI: N = 148, 0.38%, ROR = 19.21, IC = 4.23) are PTs common to both drugs. In addition, the effects of pneumonia (not in the top 10, but we were interested) were (BUD/GLY/FOR: N = 134, 0.82%, ROR = 1.64, IC = 0.71; FF/UMEC/VI: N = 738, 1.89%, ROR = 3.54, IC = 1.82), respectively. Urine flow decreased (BUD/GLY/FOR: N = 5, 0.031%, ROR = 11.47, IC = 3.54; FF/UMEC/VI: N = 19, 0.049%, ROR = 17.75, IC = 4.12) belongs to the category of renal and urinary disorders, which is correlated in both drugs. In Investigations, BUD/GLY/FOR was associated with decreased height (N = 38, 0.23%, ROR = 18.48, IC = 4.19). In Injury, poisoning, and procedural complications, BUD/GLY/FOR has a PT for lip injury (N = 4, 0.024%, ROR = 10.83, IC = 3.42), while FF/UMEC/VI has a PT for foreign body in mouth (N = 16, 0.041%, ROR = 54.77, IC = 5.67). Supplementary Table S6 shows the number of AEs grouped by sex, age, and reporting countries at the PT level.

In addition, this research identified several PTs that had not previously received attention and existed outside the drug leaflets (DailyMed, 2024a; DailyMed, 2024b). Supplementary Table S7 showed the presence of BUD/GLY/FOR with hypoacusis (N = 62, 0.38%, ROR = 3.98, IC = 1.99). Supplementary Table S7 shows the presence of PTs in the vascular disorders category in FF/UMEC/VI, including arterial disorder (N = 4, 0.010%, ROR = 4.45, IC = 2.15), aneurysm (N = 11, 0.028%, ROR = 3.99, IC = 1.99), and arterial occlusive disease (N = 8, 0.020%, ROR = 2.12, IC = 1.08), and arteriosclerosis exists in BUD/GLY/FOR (N = 5, 0.031%, ROR = 2.42, IC = 0.12). There are PTs associated with malignancy in both FF/UMEC/VI and BUD/GLY/FOR.

In order to examine the changes in each signal over time, the present study was time-scanned for BUD/GLY/FOR and FF/UMEC/VI for pneumonia, dysphonia, oral candidiasis, aphonia, *candida* infection, body height decreased, urine, decreased, tongue coated, vocal cord disorder, and cancer. The results (Supplementary Figures S1, S2) showed unstable associations between pneumonia and BUD/GLY/FOR and unstable associations between cancer-related signals and FF/UMEC/VI and BUD/GLY/FOR. The associations of the other signals with drugs remained consistent across years.

## Discussion

To our knowledge, there are few previous articles analyzing the AE of single inhaler triple-therapy drugs. Respiratory, thoracic and mediastinal disorders, injury, poisoning and procedural complications, and eye disorders were the SOCs for which the two drugs fit the study algorithm. Device use problems are common and well-known PTs for both drugs, with BUD/GLY/FOR having a significantly higher rate of device use problems than FF/UMEC/VI. After excluding PTs for substance use problems and treatment of disease, we screened a total of 17 PTs from 7 SOCs based on the top 10 highest ICs for each drug. We also analyzed pneumonia, an important PT, in both drugs. In addition, a number of PTs that are uncommon and exist outside of the drug leaflets are included in the discussion.

BUD/GLY/FOR and FF/UMEC/VI are essential medications for COPD, with common AEs stemming from the drug itself and the form of administration. In the SOCs of injury, poisoning, and procedural complications, device use issues were common to both medications for the PT. There have been few comparative studies analyzing BUD/GLY/FOR and FF/UMEC/VI on device use issues. We found through our research that BUD/GLY/FOR had a higher incidence of equipment use problems compared to FF/UMEC/VI. BUD/GLY/FOR uses a pressurized metered-dose inhaler (pMDI). This inhaler requires hand pressure to test the patient’s hand-mouth coordination and can easily compromise efficacy due to improper handling. The dry powder inhaler (DPI) of FF/UMEC/VI is relatively simple to operate. In addition to the operation of the device, the pulmonary deposition rate of the drug cannot be ignored. The pMDI of BUD/GLY/FOR utilizes a co-suspension delivery technique that is less susceptible to delivery variability and therefore may have better lung deposition rates (Dunn et al., 2020). Usmani et al. found by computerized respiratory imaging that BUD/GLY/FOR had significantly higher drug deposition in small and large airways than FF/UMEC/VI (Usmani et al., 2023). It is necessary to choose the inhaler according to the patient’s characteristics and usage habits.

Since both drugs are inhaled from the mouth via the wrapping of the lips, they then pass through the pharynx into the lungs. Any body part in the pathway could come into contact with the drug, resulting in an adverse event. Our study identified multiple PTs in three SOCs affected by the drug inhalation pathway, including respiratory, thoracic and mediastinal disorders (laryngeal, vocal folds, and voice-related PT), gastrointestinal disorders (Oral mucosal exfoliation, coating in the mouth, and tongue coated) and injury, poisoning and procedural complications (lip injury and foreign body in mouth). The above PTs may be associated with mucosal damage to the mouth, tongue, vocal cords, and other areas caused by ICS use (Rachelefsky et al., 2007; Ozbilen et al., 2010). However, these PTs do not usually result in discontinuation of therapy (Yang et al., 2023). It may reduce the incidence of associated PTs by urging patients to rinse their mouths promptly after inhalation of medications and to reduce swallowing.

Infection is a category of adverse events that cannot be ignored in BUD/GLY/FOR and FF/UMEC/VI. Regarding infections and infestations, we found that the proportions and effect sizes of oral *Candida* infections were significantly greater in FF/UMEC/VI than in BUD/GLY/FOR. This finding fits with the study of Dekuijzen et al., who found that oral *Candida* infections were less likely to occur in BUD/GLY/FOR (Dekhuijzen et al., 2016). Few previous studies have compared the risk of pneumonia with three inhalations of a drug, so we focused on the outcomes of pneumonia across the two drugs. The results showed that there was a risk of pneumonia with both drugs, with BUD/GLY/FOR having a much smaller risk of pneumonia than FF/UMEC/VI. Pneumonia risk was significantly and positively correlated with ICS use (Rønn et al., 2023). Similar to our findings, previous studies have found that patients receiving budesonide/formoterol have a substantially lower risk of pneumonia than those receiving fluticasone/salmeterol (Janson et al., 2013; Yang et al., 2017). However, a meta-analysis showed no relevant difference in the risk of pneumonia between FF/UMEC/VI and BDP/FOR/GLY (Rogliani et al., 2022).

FF/UMEC/VI and BUD/GLY/FOR had a higher risk for two categories of SOCs, vascular disease, and neoplasms, not seen in drug leaflets or previous studies. Regarding vascular disorders, PTs with arterial disorders, aneurysms, and arterial occlusive disease were seen in FF/UMEC/VI, while the PT with atherosclerosis was seen in BUD/GLY/FOR, and the reason for this association is unclear. A previous meta-analysis showed no significant difference in cardiovascular risk between the two drugs (Rogliani et al., 2022). Our study found lung cancer-associated AEs in both FF/UMEC/VI and BUD/GLY/FOR users. In addition to lung cancer PT, thoracic cancer and brain neoplasm malignant have also been found to be associated with FF/UMEC/VI. However, the results of the time-scan plots showed that the correlation between BUD/GLY/FOR and FF/UMEC/VI and tumor was unstable across years. BUD/GLY/FOR and FF/UMEC/VI have not been reported to increase tumor risk in past large clinical trials and meta-analyses (Lipson et al., 2020; Ferguson et al., 2018; Heo, 2021; Bourdin et al., 2021). Therefore, we believe that we cannot link FF/UMEC/VI and BUD/GLY/FOR to tumor-associated PTs and that it may be the chronic inflammation of asthma or COPD that contributes to tumorigenesis.

In BUD/GLY/FOR, body height decreased is the PT that exists outside of the drug leaflets. We speculated that BUD/GLY/FOR may affect the height development of minors. Pedersen found that although budesonide affects growth rates in the first few years of treatment, children can eventually reach normal adult height (Agertoft and Pedersen, 2000). However, a meta-analysis found that adolescent patients treated with budesonide 400 μg/d for an average of 4.3 years had a mean reduction in height of 1.20 cm (Zhang et al., 2014). Another study noted that fluticasone at the same dose was less inhibitory to growth than budesonide (Axelsson et al., 2019). Supplementary Table S6 analyzes the number of occurrences of body height decreased in BUD/GLY/FOR in different age groups. Unfortunately, we cannot conclude that BUD/GLY/FOR affects the height of minors due to the fact that most of the age data are missing. The relationship between BUD/GLY/FOR and height loss needs to be further substantiated. Hypoacusis is also an uncommon AE for BUD/GLY/FOR, suggesting that BUD/GLY/FOR may affect the auditory system.

The study is a pharmacovigilance analysis based on the FDA’s Adverse Event Reporting System, which is characterized by data derived from the real world. However, there are undeniable limitations to this study. First, relying on reports initiated by patients, physicians, and pharmaceutical companies to the FDA may result in underreporting and inaccuracy of specific adverse events. Second, patients and professionals report different accuracies resulting in the possibility that some AEs may be inaccurate, which leads to some bias in the results. Thus, there are areas for improvement at the data level of uncertainty of causality and duplication of reporting (Yu et al., 2021). Third, the study could not be cross-analyzed with other drugs, which may also have caused some bias. In addition, some relevant information, such as age and sex, was missing from the data, which affected the accuracy of further subgroup analysis. Finally, due to the lack of data related to the drug beclomethasone dipropionate/formoterol fumarate/glycopyrronium bromide in the database, we compared only the drugs BUD/GLY/FOR and FF/UMEC/VI. These limitations should be considered when making clinical management and decisions regarding pharmacovigilance data for BUD/GLY/FOR and FF/UMEC/VI.

## Conclusion

Our study found that the most common AEs present on the drug leaflets for BUD/GLY/FOR and FF/UMEC/VI included device use issues, multiple pharyngeal problems, pneumonia, *candida* infections, and urinary retention. Of these, the risk of pneumonia and *candida* infection was higher for FF/UMEC/VI than for BUD/GLY/FOR, and the risk of AEs associated with device use issues was higher for BUD/GLY/FOR than for FF/UMEC/VI. Outside of the drug leaflets, both drugs were associated with a higher risk of AEs in vascular disorders, and BUD/GLY/FOR group had a higher risk of AEs in body height decreased and hypoacusis. Our study provides valuable insights into the safety of the clinical use of BUD/GLY/FOR and FF/UMEC/VI, and clinicians should remain vigilant for potential AEs.

## Data Availability

The raw data supporting the conclusions of this article will be made available by the authors, without undue reservation.

## Supplementary material

SUPPLEMENTARY FIGURE S1 Information components of BUD/GLY/FOR and FF/UMEC/VI over time in AEs with top ten IC values.

SUPPLEMENTARY FIGURE S2 Information components of BUD/GLY/FOR and FF/UMEC/VI over time in tumor-related AEs.
