# Supplementary figures and images for: High-risk adverse events in two types of single inhaler triple-therapy: a pharmacovigilance study based on the FAERS database
Source: Front Pharmacol. 2025 Jan 9;15:1460407. doi: 10.3389/fphar.2024.1460407 (PMC11754260; doi:10.3389/fphar.2024.1460407)

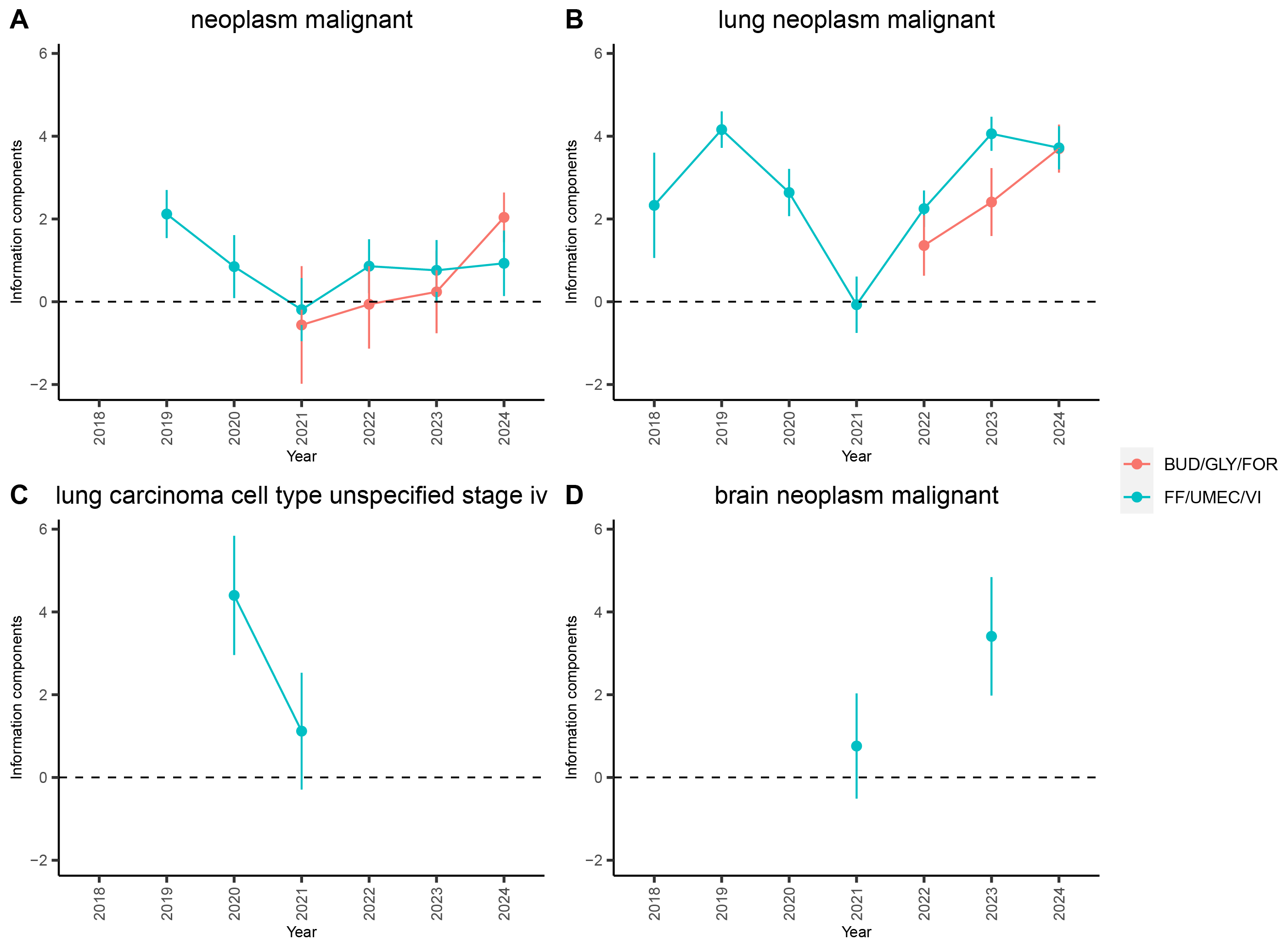

Supplement: Supplementary file 1 [file Image2.tif]

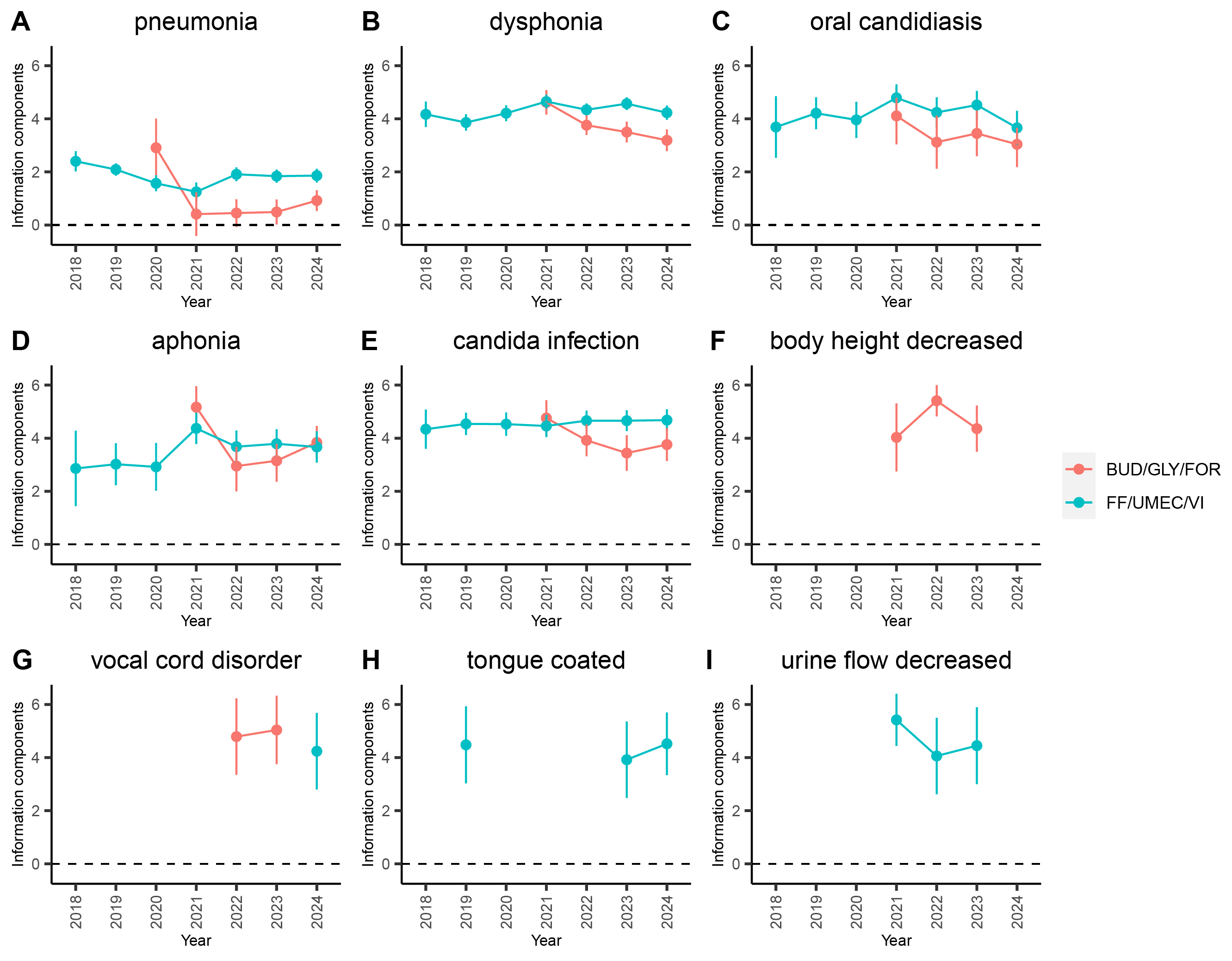

Supplement: Supplementary file 2 [file Image1.tif]
